# Overexpression of apolipoprotein A-I alleviates endoplasmic reticulum stress in hepatocytes

**DOI:** 10.1186/s12944-017-0497-3

**Published:** 2017-06-02

**Authors:** Qing Guo, Can Zhang, Yutong Wang

**Affiliations:** Department of Cell Biology, Municipal Laboratory for Liver Protection and Regulation of Regeneration, School of Basic Medical Sciences, Capital Medical University, 10 You An Men Wai Xi Tou Tiao, Beijing, 100069 China

**Keywords:** Apolipoprotein A-I, Endoplasmic reticulum stress, Sterol regulatory element binding protein, Non-alcoholic fatty liver disease

## Abstract

**Background:**

Abnormal lipid metabolism may contribute to an increase in endoplasmic reticulum (ER) stress, resulting in the pathogenesis of non-alcoholic steatohepatitis. Apolipoprotein A-I (apoA-I) accepts cellular free cholesterol and phospholipids transported by ATP-binding cassette transporter A1 to generate nascent high density lipoprotein particles. Previous studies have revealed that the overexpression of apoA-I alleviated hepatic lipid levels by modifying lipid transport. Here, we examined the effects of apoA-I overexpression on ER stress and genes involved in lipogenesis in both HepG2 cells and mouse hepatocytes.

**Methods:**

Human apoA-I was overexpressed in HepG2 hepatocytes, which were then treated with 2 μg/mL tunicamycin or 500 μM palmitic acid. Eight-week-old male apoA-I transgenic or C57BL/6 wild-type mice were intraperitoneally injected with 1 mg/kg body weight tunicamycin or with saline. At 48 h after injecting, blood and liver samples were collected.

**Results:**

The overexpression of apoA-I in the models above resulted in decreased protein levels of ER stress makers and lipogenic gene products, including sterol regulatory element binding protein 1, fatty acid synthase, and acetyl-CoA carboxylase 1. In addition, the cellular levels of triglycerides and free cholesterol also decreased. Some of gene products which are related to ER stress-associated apoptosis were also affected by apoA-I overexpression. These results suggested that apoA-I overexpression could reduce steatosis by decreasing lipid levels and by suppressing ER stress and lipogenesis in hepatocytes.

**Conclusion:**

ApoA-I expression could significantly reduce hepatic ER stress and lipogenesis in hepatocytes.

## Background

Non-alcoholic fatty liver disease (NAFLD) is a chronic metabolic disease characterised by fatty infiltration of the liver in the absence of chronic alcohol consumption and macrovesicular steatosis. NAFLD is strongly associated with obesity, insulin resistance, metabolic syndrome, and type II diabetes [1–3]. Some patients may even develop non-alcoholic steatohepatitis (NASH), which is characterised by superimposed ballooned hepatocytes, Mallory bodies, and lobular inflammatory cell infiltration. Increasing evidence has demonstrated that endoplasmic reticulum (ER) stress may represent an intrinsic second hit that triggers NASH in the steatotic liver [4–6].

ER is an intracellular organelle responsible for the folding of membrane and secretory proteins, the synthesis of lipids and sterols, and several important cellular functions, including Ca^2+^storage and cell signalling [7, 8]. Under certain physiological, pharmacological, and pathological conditions, including free cholesterol accumulation in the ER, impaired ER functions and protein folding capacity may result in ER stress or unfolded protein response, which plays an important role in NAFLD [9, 10]. ER stress rapidly induces the cleavage of the precursor form of sterol regulatory element binding protein (SREBP) 1c as well as the expression of SREBP-1c target genes [4].If none of the adaptive measures is able to resolve the sustained ER stress, cell death by apoptosis is initiated [11].

Apolipoprotein A-I (apoA-I), the primary protein component of high density lipoproteins, functions by accepting cellular cholesterol and phospholipids that are transported by the ATP-binding cassette transporter A1 (ABCA1) during the initial step of reverse cholesterol transport [12]. D4F, an apoA-I mimetic peptide, may inhibit ER stress induced by oxidised low density lipoproteins in macrophages [13]. Furthermore, apoA-I mimic peptide can also prevent inflammatory reactions and reduce atherosclerosis in mice [14]. However, it remains unclear whether the effect of apoA-I is strictly due to the function of reverse cholesterol transport or due to interactions with circulating proteins.

Previous studies have suggested that apoA-I expression promotes the clearance of reverse cholesterol transport from hepatocytes, reduces hepatic lipid accumulation, and suppresses fatty acid synthesis as well as the expression of cyclooxygenase-2 [15–17]. In the current study, we examined the effects of apoA-I on ER stress in the human hepatic cell line HepG2, as well as in wild type and apoA-I transgenic C57BL/6 J mice. We showed that overexpression of apoA-I significantly suppressed the expression of ER stress-related markers both in vitro and in vivo. In addition, apoA-I expression also reduced hepatic apoptosis in the HepG2 cells.

## Methods

### Chemicals, reagents and cells

HepG2 cells were obtained from the American Type Culture Collection (ATCC). Dulbecco’s modified Eagle’s medium (DMEM) and foetal bovine serum (FBS) were acquired from HyClone (Logan, UT, USA). FuGENE HD was obtained from Promega (Madison, WI, USA). Free cholesterol and triglyceride quantification kits were purchased from Applygen Technologies (Beijing, China). Tunicamycin (TM), palmitic acid (PA), and fatty acid-free bovine serum albumin (BSA) were obtained from Sigma (St. Louis, MO, USA). Antibodies against glucose-regulated protein 78 (GRP78) and glyceraldehyde 3-phosphatedehydrogenase (GAPDH) were purchased from Sigma and Shanghai Kang Chen Biotech (Shanghai, China), respectively. Antibodies against apoA-I and acetyl-CoA carboxylase1 (ACC1) were purchased from Cell Signaling Technology (Danvers, MA, USA). Antibodies against apoA-I, C/EBP homologous protein (CHOP), PKR-like ER kinase (PERK), SREBP-1, fatty acid synthase (FAS), Bax, Bcl2, and caspase-3 were purchased from Santa Cruz Biotechnology (Santa Cruz, CA, USA).

### Cell culture and plasmid transfection

HepG2 cells were maintained in DMEM containing 10% FBS or were incubated in DMEM containing 1% FBS. pcDNA3.0/apoA-I and the control plasmid were transfected into HepG2 cells using FuGENE HD as described in the manufacturer’s protocol. Washed cells were incubated for 24 h with medium containing 1% FBS in the presence or absence of 2 μg/mL TM or 500 μM PA.

### Determination of fatty cholesterol and triglycerides

Intracellular and liver triglyceride levels were assayed using a triglyceride assay kit (Applygen Technologies). Intracellular and hepatic free cholesterol levels were measured using an assay kit for free cholesterol (Applygen Technologies) according to the manufacturer’s recommended protocol.

### Western blot

Cells were washed with PBS and lysed using RIPA buffer containing protease inhibitors. Cell debris and insoluble proteins were removed by centrifugation at 12,500 rpm for 10 min at 4 °C. Equal amounts of proteins were resolved by SDS-PAGE. The expression levels of apoA-I, GRP78, P-PERK, PERK, CHOP, SREBP-1, FAS, ACC1, Bax, Bcl2, and caspase-3 were determined by Western blot analysis using the appropriate antibodies.

### Caspase activity assay

Intracellular caspase-4 activity levels were detected by the caspase-4 colorimetric assay kit (BioVision, Mountain View, CA, USA). Intracellular caspase-3/7 activity levels were measured using the Caspase-Glo 3/7 assay kit (Promega, Madison, WI, USA) according to the manufacturer’s instructions.

### Animal studies

ApoA-I transgenic (Tg) mice were purchased from the Jackson Laboratory and bred at the Department of Laboratory Animal Science in Capital Medical University. Eight-week-old male Tg and C57BL/6 wild-type (WT) mice were intraperitoneally injected with 1 mg/kg body weight TM or with saline. At 48 h after injecting, blood and liver samples were collected. Experimental research on mice has been approved by the animal ethics committee in Capital Medical University.

### Histological analysis of tissue samples

After the mice were sacrificed, the livers were collected, and they were stored at −80 °C, fixed in 10% formalin, or prepared for frozen sections. Five micrometer-thick sections were obtained from formalin-fixed paraffin-embedded tissues for histological analyses. Conventional haematoxylin and eosin (H&E) histological staining and Oil Red O staining were performed in order to evaluate the microscopic morphology of the liver tissue samples. Ten micrometer-thick sections were obtained from frozen tissue for Oil Red O staining in order to evaluate the lipid levels in liver.

### Statistical analysis

The results of multiple observations are presented as the mean ± SEM. The data were analysed with the statistics software GraphPad Prism 5 (GraphPad Software, La Jolla, CA, USA) by a nonparametric analysis of variance test. Differences were considered significant if *p* < 0.05.

## Results

### Evaluation of TM-induced ER stress in HepG2 cells

**Fig. 1:**
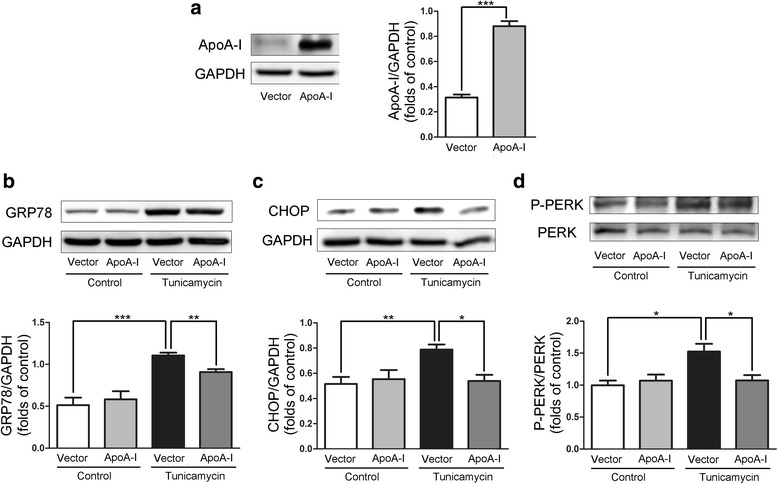
ApoA-I overexpression reduces TM-induced ER stress in HepG2 cells.HepG2 cells were transfected with pcDNA3.0 alone or with pcDNA3.0/apoA-I plasmid using the FuGENE HD transfection reagent. Twenty-four hours after transfection, the transfected cells were incubated for 24 h in DMEM in the presence or absence of 2 μg/mL TM, and total cellular proteins were isolated and analysed for apoA-I (**a**), GRP78 (**b**), CHOP (**c**) and P-PERK (**d**) by Western blotting. The results are representative of three independent experiments and are presented as the mean ± SEM. *P < 0.05 versus control. **P < 0.01 versus control. ***P < 0.001 versus control

**Fig. 2:**
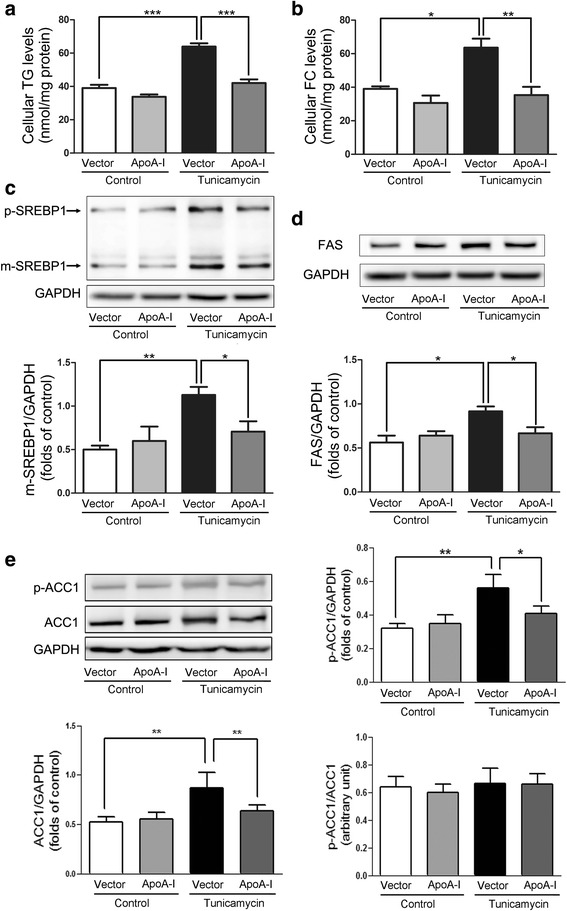
ApoA-I overexpression downregulates lipid accumulation and alters SREBP-1, FAS and ACC1 protein expression in HepG2 cells. HepG2 cells were transfected with pcDNA3.0 alone or with pcDNA3.0/apoA-I plasmid using the FuGENE HD transfection reagent. Twenty-four hours after transfection, the transfected cells were incubated for 24 h in DMEM in the presence or absence of 2 μg/mL TM, and levels of cellular triglycerides (**a**) as well as free cholesterol (**b**) were measured. In addition, total cellular proteins were isolated and analysed for p-SREBP1, m-SREBP1 (**c**), FAS (**d**) and p-ACC1/ACC1 (**e**) by Western blotting. The results are representative of three independent experiments and are presented as the mean ± SEM. *P < 0.05 versus control. **P < 0.01 versus control. ***P < 0.001 versus control

TM induces ER stress in HepG2 cells [18] and subsequently reduces apoA-I expression [19]. We increased apoA-I expression levels in TM-treated HepG2 cells by transfecting the cells with plasmids expressing apoA-I (Fig. 1a). The overexpression of apoA-I resulted in a significant decrease with 2 μg/mL TM-induced ER stress for 24 h, as measured by the expression of ER stress markers: GRP78 (Fig. 1b), CHOP (Fig. 1c) and P-PERK/PERK (Fig. 1d). These results support the hypothesis that apoA-I overexpression can reduce the ER stress induced by TM. It has been previously reported that TM can also induce SREBPs and consequently increase hepatic biosynthesis as well as the uptake of cholesterol and triglycerides, leading to hepatic steatosis [4]. Thus, the expression levels of apoA-I may cause a significant reduction in the cellular lipid accumulation induced by TM. To test this hypothesis, we altered apoA-I expression levels in TM-treated HepG2 cells by transfecting the cells with apoA-I-expressing plasmid. As expected, apoA-I overexpression caused a significant decrease in the levels of cellular triglycerides and free cholesterol (Fig. 2a, b). Furthermore, upon expression of apoA-I, the levels of mature SREBP1 (Fig. 2c), FAS (Fig. 2d) and ACC1 (Fig. 2e) decreased significantly, indicating a reduction in lipid synthesis. These data further support the hypothesis that apoA-I overexpression can reduce ER stress induced by TM.

### Evaluation of PA-induced ER stress inHepG2 cells

**Fig. 3:**
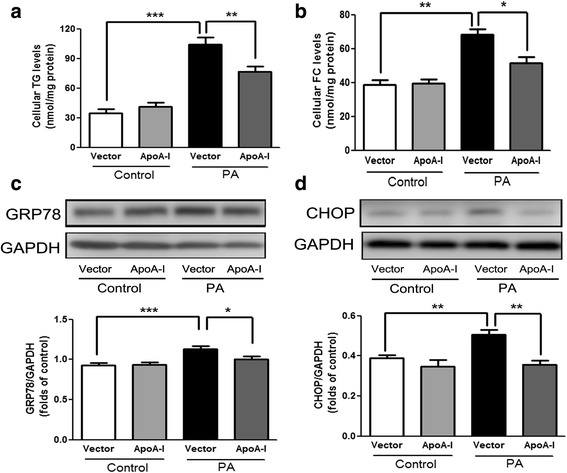
ApoA-I overexpression reduces PA-induced lipid accumulation and ER stress in HepG2 cells.HepG2 cells were transfected with pcDNA3.0 alone or the pcDNA3.0/apoA-I plasmid using the FuGENE HD transfection reagent. Twenty-four hours after transfection, the transfected cells were incubated for 24 h in DMEM with 5 mM BSA in the presence or absence of 500 μM PA, and levels of cellular triglycerides (**a**) as well as free cholesterol (**b**) were measured. In addition, total cellular proteins were isolated and analysed for GRP78 (**c**) and CHOP (**d**) by Western blotting. The results are representative of three independent experiments and are presented as the mean ± SEM. *P < 0.05 versus control. **P < 0.01 versus control. ***P < 0.001 versus control

**Fig. 4:**
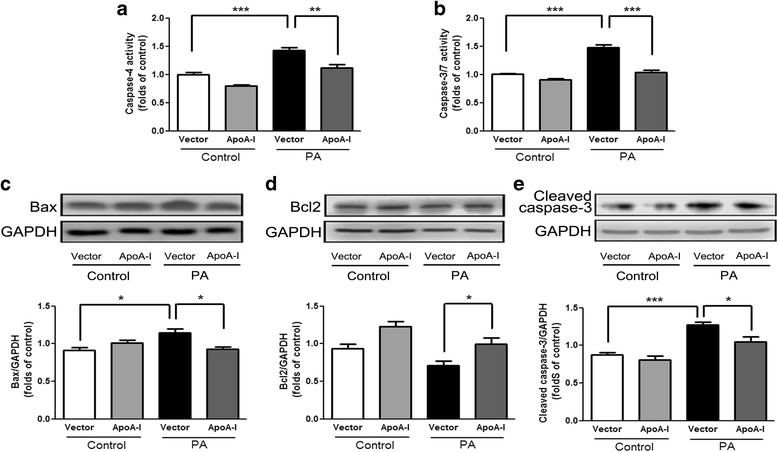
ApoA-I overexpression downregulates PA-inducedapoptosis in HepG2 cells. HepG2 cells were transfected with pcDNA3.0 alone or the pcDNA3.0/apoA-I plasmid using the FuGENE HD transfection reagent. Twenty-four hours after transfection, the transfected cells were incubated for 24 h in DMEM with 5 mM BSA in the presence or absence of 500 μM PA, and activities of cellular caspase-4 (**a**) as well as caspase-3/7 (**b**) were measured. In addition, total cellular proteins were isolated and analysed for Bax (**c**), Bcl2 (**d**), and cleaved caspase-3 (**e**) by Western blotting. The results are representative of three independent experiments and are presented as the mean ± SEM. *P < 0.05 versus control. **P < 0.01 versus control. ***P < 0.001 versus control

To determine whether apoA-I overexpression can reduce ER stress induced by another mechanism, we tested the effects of apoA-I overexpression in PA-induced ER stress in HepG2 cells. Similar to the results obtained from the TM-induced ER stress, apoA-I overexpression caused a significant decrease in cellular triglycerides and free cholesterol (Fig. 3a, b). In addition, the overexpression of apoA-I resulted in a significant decrease in PA-induced ER stress, as measured by the expression of GRP78 (Fig. 3c) and CHOP (Fig. 3d). Cytochrome c from the mitochondria, mediated by Bax and Bcl2, has been implicated in ER stress-associated apoptosis [20]. Our results showed that overexpression of apoA-I significantly reduced the activities of caspase-4 (Fig. 4a), which is a caspase associated with ER stress [21]. Overexpression of apoA-I also reduced the levels of apoptotic regulator Bax and increased the levels of anti-apoptotic regulator Bcl2 (Fig. 4c, d). Consequently, the levels of cleaved caspase-3 (Fig. 4b) and the activities of caspase-3/7 (Fig. 4e) were also decreased by apoA-I overexpression. These data suggest that apoA-I expression can reduce ER stress-associated apoptosis.

### Evaluation of ER stress in mice

**Fig. 5:**
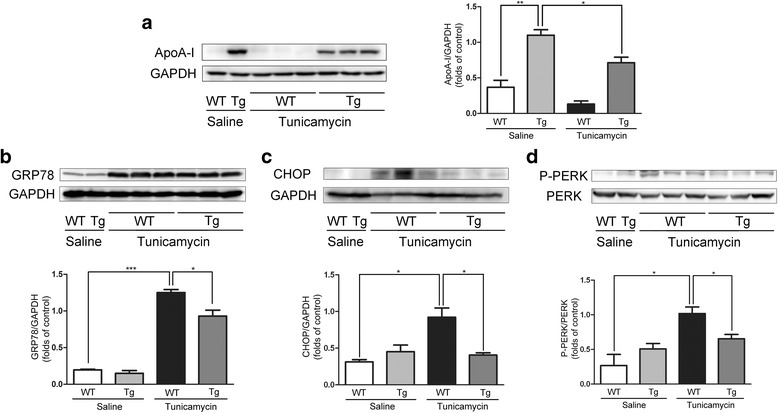
ApoA-I overexpression prevents ER stress in mice by TM challenge. Wild-type and apoA-I-Tg mice were challenge by TM through intraperitoneal injections. After 48 h of TM (1 mg/kg body weight) challenge, hepatic proteins were isolated and analysed for apoA-I (**a**), GRP78 (**b**), CHOP (**c**) and P-PERK (**d**) by Western blotting. The results are representative of three independent experiments and are presented as the mean ± SEM. *P < 0.05 versus control. **P < 0.01 versus control. ***P < 0.001 versus control

**Fig. 6:**
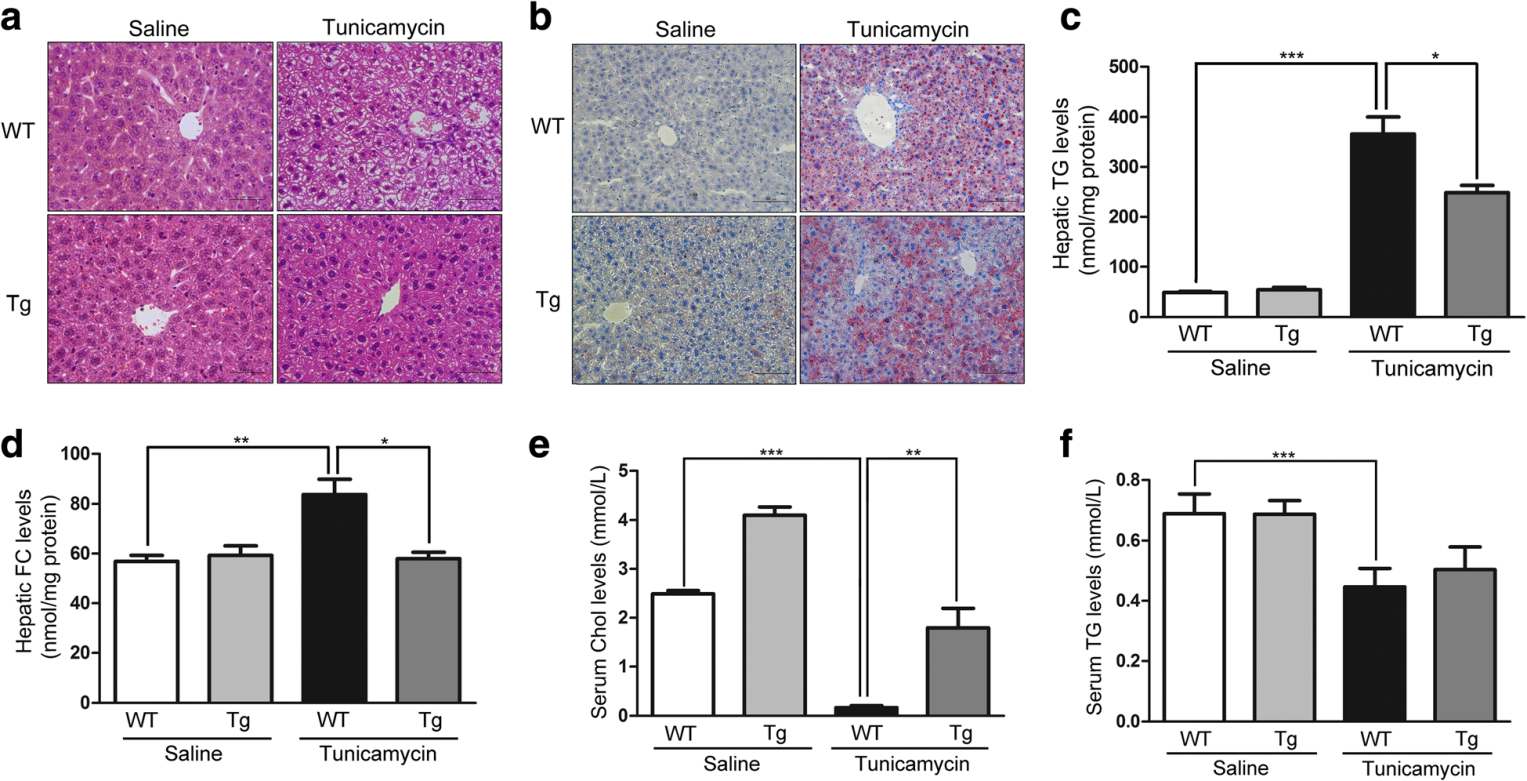
ApoA-I overexpression reduces hepatic steatosis in mice by TM challenge. H&E (**a**) and Oil Red O (**b**) staining for wild-type and ApoA-I-Tg mice injected with TM (1 mg/kg body weight). Levels of hepatic triglyceride (**c**) and free cholesterol (**d**) were measured as described in the “Methods”. Serum cholesterol (**e**) and triglyceride (**f**) levels were detected with Roche Module P-800. The results are representative of three independent experiments and are presented as the mean ± SEM. *P < 0.05 versus control. **P < 0.01 versus control. ***P < 0.001 versus control

**Fig. 7:**
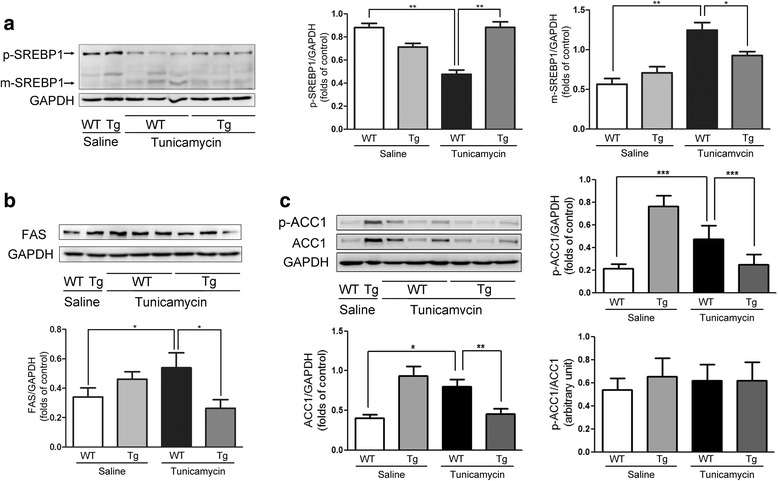
ApoA-I overexpression decreases the expression of m-SREBP1, FAS and ACC1 in mice by TM challenge. Wild-type and ApoA-I-Tg mice were challenge with TM through intraperitoneal injection. After 48 h of TM (1 mg/kg body weight) challenge, hepatic proteins were isolated and analysed for m-SREBP1(**a**), FAS (**b**) and p-ACC1/ACC1(**c**) by Western blotting. The results are representative of three independent experiments and are presented as the mean ± SEM. *P < 0.05 versus control. **P < 0.01 versus control

TM has been shown to be an efficient pharmacological tool to induce acute ER stress in the liver in vivo [22, 23]. To study the effects of apoA-I on the liver pathophysiology after pharmacologically induced ER stress, we challenged wild-type and apoA-I-Tg mice with TM through intraperitoneal injections. At 48 h after TM treatment, the levels of apoA-I were reduced significantly (Fig. 5a). In addition, the expression of GRP78 (Fig. 5b), CHOP (Fig. 5c), and P-PERK (Fig. 5d) was detected by Western blot. These results indicate that apoA-I significantly suppressed these ER stress-related markers. These data further support the hypothesis that apoA-I overexpression can reduce lipid accumulation induced by TM in mice. It has been reported that overexpression of apoA-I in mice can reduce hepatic lipid levels with methionine and choline deficient diets [15] and that lipid toxicity may play a key role in ER stress [24]. After 48 h of TM challenge, liver tissues and blood plasma samples from WT and apoA-I-Tg mice were collected for histological analysis and lipid profiling. Consistent with the results from cultured cells, expression of apoA-I in vivo significantly reduced hepatic lipid deposition induced by TM, as shown by the H&E (Fig. 6a) and Oil Red O (Fig. 6b) stainings as well as the levels of hepatic triglycerides (Fig. 6c) and free cholesterol (Fig. 6d). Consistent with a previous report [25], compared to the WT mice, apoA-I-Tg mice showed higher levels of serum cholesterol (Fig. 6e) without any alteration in the levels of serum triglycerides (Fig. 6f). Collectively, these data indicate that overexpression of apoA-I in the apoA-I-Tg mice had reduced hepatic steatosis upon TM challenge, in contrast with the WT mice, providing evidence that apoA-I reduced the lipid toxicity induced by TM in mice. ER stress may also cause hepatic steatosis and upregulate de novo lipogenesis through the activation of SREBP1c in the liver [26]. To determine the effects of apoA-I on the hepatic lipid metabolism in TM-treated mice, we examined the activation of hepatic SREBP1, the key transcriptional regulator of de novo lipogenesis. Compared with WT mice, apoA-I-Tg mice showed a dramatic decrease in the level of cleaved SREBP1 protein in the liver tissues. Indeed, the levels of SREBP1 precursors were diminished, while mature forms of SREBP1 accumulated in the tissues (Fig. 7a). Moreover, this also resulted in a decrease in the expression levels of some lipogenic genes in the liver of apoA-I-Tg mice, including FAS (Fig. 7b) and ACC1 (Fig. 7c). These results provide evidence that apoA-I inhibits ER stress-induced lipid accumulation through the inhibition of the SREBP-1 pathway.

## Discussion

Previous studies have shown that ER stress may contribute to the development of NAFLD. This may occur due to the dysregulation of the endogenous sterol response pathway induced by ER stress, which can cause increased hepatic biosynthesis and uptake of cholesterol and triglycerides [4]. In particular, ER stress decreases the protein levels of hepatic ABCA1 and apoA-I [19, 27, 28], which may result in the impaired transport of cholesterol and phospholipids in the liver, thereby leading to NAFLD. Moreover, ER stress has been detected in the adipose tissue and liver of patients with NAFLD [29, 30]. Our previous studies have revealed that increased expression of apoA-I effectively regulates hepatic fatty acid and have a beneficial effect on NASH [15]. Here, we provide further evidence for the beneficial effects of apoA-I on ER stress-induced hepatic steatosis. HepG2 cells transfected with apoA-I showed significant protection against ER stress as well as ER stress-mediated lipid accumulation and apoptosis. In addition, apoA-I-Tg mice displayed a clear capability to inhibit ER stress and hepatic steatosis induced by TM. Finally, we showed that apoA-I may reduce the levels of some lipogenic genes, including SREBP-1, FAS, and ACC1, which play crucial roles in lipid accumulation and ER stress, in both HepG2 cells and mice. This observation revealed an association between hepatic apoA-I, ER stress, and SREBP-1, which further suggests that upregulation of apoA-I can improve NAFLD.

Recent data have suggested that accumulation of free cholesterol is relevant to the pathogenesis of NAFLD/NASH [31, 32]. Free cholesterol accumulation leads to liver injury through the activation of intracellular signalling pathways in Kupffer cells, stellate cells, and hepatocytes. In addition, free cholesterol accumulation in liver mitochondria induces mitochondrial dysfunction, which results in increased production of reactive oxygen species (ROS), and triggers the unfolded protein response in the ER, thereby causing ER stress and apoptosis [33]. ApoA-I can accept cellular cholesterol and phospholipids transported by ABCA1 at the initial step of reverse cholesterol transport. In addition, our previous studies demonstrated that the upregulation of apoA-I can reduce ROS [17]. Therefore, apoA-I attenuates ER stress by modulating free cholesterol and ROS.

Increasing evidence has demonstrated that ER stress plays an important role in hepatic SREBP1c activation [34]. According to an earlier report, ER stress was associated with hepatic SREBP1c activation [4]. A recent study of TM-treated mice found that ER stress can lead to a dramatic conversion of the precursors of SREBP1c into their mature form in the liver [26]. Consequently, the mature form of SREBP-1c may activate lipogenic genes such as FAS and ACC1.We have shown that apoA-I decreases the expression levels of m-SREBP-1, FAS, and ACC1 induced by TM, and this effect may be due to the reduction of ER stress. However, under normal physiological conditions, apoA-I overexpression may result in the upregulation of m-SREBP1, FAS and ACC1 expression to compensate for the cellular lipids removed by apoA-I. The effects apoA-I may also be due to the stimulation of other intracellular signalling pathways. For example, it has been shown that apoA-I increases mitochondrial biogenesis through AMP-activated protein kinase (AMPK) [35], and fasting-induced hepatic steatosis can be exacerbated by the impairment of AMPK [36] and rescued by AMPK activator [37], suggesting multiple functions of apoA-I in NASH.

This study has important therapeutic implications for the treatment of NASH. By modulating apoA-I expression, both the levels of ER stress as well as expression of lipogenic genes were significantly reduced. These effects may contribute to the removal of cholesterol and phospholipids. In addition, overexpression of apoA-I may also suppress fatty acid synthesis through a reduction in LXR ligand levels [16]. As a result, stimulation of the ABCA1/apoA-I pathway can decrease the lipid levels in hepatocytes, which in turn causes a decrease in ER stress levels as well as the expression levels of lipogenic genes, eventually resulting in a reduction in hepatic steatosis. Therefore, understanding the mechanisms of these processes would be useful for designing apoA-I-based therapeutic interventions that could enhance the activity of hepatic lipid removal and thus prevent the development of NASH.

## Conclusion

ApoA-I expression could significantly reduce hepatic ER stress and lipogenesis in hepatocytes.

## Abbreviations

ABCA1: ATP-binding cassette transporter A1
ACC1: Acetyl-CoA carboxylase 1
apoA-I: Apolipoprotein A-I
BSA: Bovine serum albumin
CHOP: C/EBP homologous protein
DMEM: Dulbecco’s modified Eagle’s medium
ER: Endoplasmic reticulum
FAS: Fatty acid synthase
FBS: Foetal bovine serum
GAPDH: Glyceraldehyde 3-phosphate dehydrogenase
GRP78: Glucose-regulated protein 78
H&E: Haematoxylin and eosin
NAFLD: Non-alcoholic fatty liver disease
NASH: Non-alcoholic steatohepatitis
PERK: PKR-like ER kinase
ROS: Reactive oxygen species
SREBP: Sterol regulatory element binding protein
